# Comparative Evaluation of QQ Media Materials for MBR Applications: An Environmental Footprint Approach in Urban Wastewater Treatment Plants

**DOI:** 10.3390/membranes16050161

**Published:** 2026-04-30

**Authors:** Semanur Korkusuz-Soylu, Rabia Ardic-Demirbilekli, Merve Yilmaz, Ismail Koyuncu, Borte Kose-Mutlu

**Affiliations:** 1 Environmental Engineering Department, Istanbul Technical University, 34469 Istanbul, Türkiye; korkusuz16@itu.edu.tr (S.K.-S.); ardic20@itu.edu.tr (R.A.-D.); durmusmer@itu.edu.tr (M.Y.); koyuncu@itu.edu.tr (I.K.); 2 National Research Center on Membrane Technologies, Istanbul Technical University, 34469 Istanbul, Türkiye

**Keywords:** carbon footprint, membrane bioreactors, membrane fouling mitigation, quorum-quenching, urban wastewater treatment plant, water footprint

## Abstract

Urban wastewater treatment plants face increasing challenges in mitigating environmental impacts while achieving high treatment efficiency. This study explores the optimization of quorum-quenching (QQ) media materials for scalable membrane bioreactor (MBR) applications, focusing on their potential to reduce operational footprints and enhance sustainability. Six immobilization media were evaluated—sodium alginate (SA), polyvinyl alcohol (PVA) beads (P), magnetic beads (M), chitosan magnetic beads (CM), polymer-coated beads (PS), and flat media (SAP)—using a multi-criteria decision analysis (MCDA) framework. Key parameters, including porosity, mechanical strength, quorum-quenching activity, and durability in sludge, were quantitatively weighted according to their operational significance. SA demonstrated the most balanced performance, exhibiting superior durability and cost-effectiveness, whereas SAP showed potential in applications prioritizing high porosity and enhanced QQ activity. The incorporation of QQ media led to a significant reduction in membrane fouling, chemical consumption, and energy consumption in pilot-scale MBR systems. Ecological footprint assessment revealed a 15% reduction in indirect blue water footprints and a 20% decrease in Scope 2 carbon emissions, attributable to reduced operational energy demands. These findings highlight the efficacy of QQ media in improving MBR performance and advancing system-level sustainability. Overall, this study highlights the critical importance of material engineering and ecological footprint integration in the development of next-generation urban wastewater treatment technologies.

## 1. Introduction

Water resources are progressively diminishing due to the rapid global population growth [1]. As water scarcity intensifies, the treatment of wastewater to meet discharge standards and its reuse as an alternative resource have become increasingly important [2,3]. Among the available treatment technologies, the membrane bioreactor (MBR) process has emerged as a promising approach. MBR systems integrate the activated sludge process with membrane separation, offering a hybrid treatment configuration [4]. While the operational principle is similar to that of conventional activated sludge systems, MBR technology eliminates the need for secondary clarification and tertiary filtration processes, such as sand filtration [5]. Despite its advantages, membrane biofouling remains a critical limitation, leading to increased operational costs and reduced treatment efficiency [6]. Biofouling occurs when retained contaminants and microorganisms in the mixed liquor accumulate on the membrane surface, resulting in a decline in permeate flux under constant transmembrane pressure (TMP) [7,8]. Strategies based on quorum-sensing (QS) disruption and quorum quenching (QQ) have been proposed to mitigate membrane biofouling and extend membrane lifespan [9]. QS refers to cell-to-cell communication mediated by signaling molecules, enabling bacterial populations to coordinate collective behavior [10,11]. Signal production and accumulation are directly correlated with cell density, and effective communication occurs only after a threshold concentration is reached [12]. Once this threshold is exceeded, signaling molecules bind to specific receptors, triggering gene expression and coordinated cellular responses [13]. This communication mechanism promotes biofilm formation through bacterial aggregation, which is a key contributor to membrane biofouling [14]. The QQ approach aims to disrupt bacterial communication pathways and regulate microbial activity [15]. Three primary mechanisms have been identified for interrupting QS signaling: (i) inhibition of signal synthesis in the sender cell, (ii) interference with signal perception, and (iii) degradation of signaling molecules in the environment [9]. A wide range of QQ strategies and immobilization media have been investigated in the literature. Although research on quorum sensing and quorum quenching in membrane bioreactor systems has expanded significantly since 2009—particularly in the last five years—existing studies remain fragmented, with only a limited number of pilot-scale applications and no reported full-scale implementations. This gap highlights the need for integrated, application-oriented evaluations addressing key challenges such as QQ stability, long-term microbial activity, and cost-effective scale-up strategies to enable the transition from laboratory research to practical QQ-MBR systems [16].

Among QQ strategies, enzymatic QQ represents one of the earliest and most extensively studied approaches, followed by alternatives such as acyl homoserine lactone (AHL)-based QQ bacteria, fungal QQ systems, and photolytic QQ mechanisms [16,17]. QQ applications can be implemented using various immobilization media. Literature surveys indicate that cell-entrapping beads (CEBs) and microbial vessels (MVs) are among the most commonly employed systems. In addition, the rotating microbial carrier frame (RMCF) has been proposed as an alternative immobilization configuration [18,19]. Other approaches include sheet-based systems [20,21] and co-culture immobilized QQ filter membranes, along with their modified and optimized carrier designs [22]. Numerous studies have focused on bacterial immobilization techniques to enhance QQ efficiency due to their favorable size, geometry, and operational flexibility [12,23].

This study aims to systematically evaluate and compare different immobilization media for quorum-quenching applications in membrane bioreactor systems, with a particular focus on their suitability for large-scale implementation. To this end, six immobilization media with distinct structural configurations (bead and flat forms) were developed and assessed under consistent experimental conditions. The study specifically investigates the effectiveness of the signal molecule degradation pathway (mechanism iii) using *Rhodococcus* sp. BH4, while integrating key performance indicators such as mechanical stability, durability in sludge environments, and quorum-quenching efficiency. Furthermore, this work extends beyond conventional material evaluation by incorporating a multi-criteria decision analysis (MCDA) framework and 1ecological footprint assessment to identify optimal media from both operational and sustainability perspectives. Through this comprehensive approach, the study seeks to bridge the gap between laboratory-scale research and practical MBR applications, providing guidance for the selection, design, and scale-up of QQ-based immobilization media in urban wastewater treatment systems.

## 2. Materials and Methods

### 2.1. Reagents

N-octanoyl-L-homoserine lactone (C8-HSL) was supplied by Cayman Chemical (Ann Arbor, MI, USA) and stored at −20 °C prior to use. Sodium alginate, acetic acid, Luria–Bertani (LB) medium, chitosan, sodium hydroxide, and polyvinyl alcohol were purchased from Sigma-Aldrich (Darmstadt, Germany). Ammonia, boric acid, calcium chloride, and N-methyl-2-pyrrolidone (NMP) were obtained from Merck (Darmstadt, Germany). Iron(III) chloride hexahydrate was supplied by Thermo Scientific (Waltham, MA, USA), while iron(II) chloride tetrahydrate was purchased from Honeywell (Charlotte, NC, USA). Glycerol was obtained from Tekkim (Istanbul, Türkiye). Polyethersulfone (Veradel PES 3000P, molecular weight: 60,000 g·mol^−1^) was procured from Solvay Specialty Polymers (Istanbul, Türkiye) *Rhodococcus* sp. BH4 cultures were maintained as glycerol stocks from previous studies and reactivated prior to experimental use.

### 2.2. QQ Bacteria: Rhodococcus sp. BH4

*Rhodococcus* sp. BH4 was originally isolated from activated sludge in a previous study conducted by Köse-Mutlu [18]. Since its isolation, the strain has been preserved as glycerol stocks at −20 °C and routinely used in subsequent experiments. All experimental procedures were conducted under aseptic conditions using sterilized materials and equipment. Sterilization was performed using an autoclave (Astell, Istanbul, Türkiye), where all media and glassware were sterilized prior to use. To maintain sterility during handling, all procedures were carried out in a Class II Type A2 laminar flow cabinet (ESCO, Labculture, Friedberg, Germany). Bacterial growth was performed using both liquid and solid culture media [18]. The liquid medium was prepared by dissolving Luria–Bertani (LB) broth in distilled water, whereas the solid medium was obtained by supplementing LB medium with agar at appropriate concentrations. Prior to inoculation, the stock culture was thawed to room temperature and transferred into liquid medium in sterile Falcon tubes. The inoculated cultures were incubated in a rotary shaker (Nüve, Istanbul, Türkiye) at 30 °C and 160 rpm for 24 h to promote bacterial growth. Following incubation, a sample from the liquid culture was aseptically streaked onto agar plates to obtain isolated colonies. The plates were incubated at 30 °C for 24 h, and colony morphology was examined to confirm the absence of contamination. Pure cultures were subsequently propagated for further experimental use. For long-term storage, bacterial cultures were mixed with glycerol in eppendorf tubes and preserved at −20 °C as stock cultures.

### 2.3. Manufacturing of the Immobilization Media

**Figure 1:**
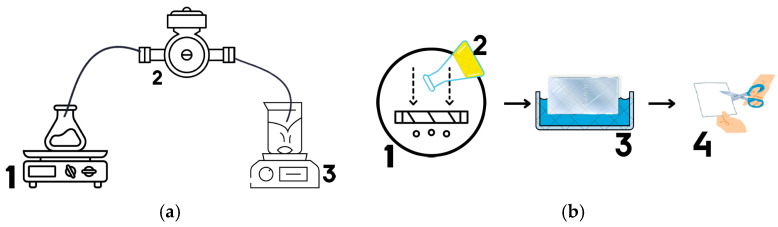
Schematic illustration of immobilization media fabrication: (**a**) bead-type media production (1: bead solution; 2: peristaltic pump; 3: bead-forming solution) and (**b**) flat-sheet media fabrication (1: membrane casting device; 2: casting solution; 3: coagulation bath; 4: cutting and application of the fabricated media).

Within the scope of this study, six distinct immobilization media were fabricated, and the production methodology is schematically presented in Figure 1.

#### 2.3.1. Sodium Alginate Beads

Sodium alginate beads were prepared following the method described by Köse-Mutlu et al. [18]. A 4% (*w*/*v*) sodium alginate solution (100 mL) and a 3% (*w*/*v*) CaCl_2_ solution (300 mL) were prepared. Due to the tendency of alginate to form aggregates in aqueous solutions, the polymer was gradually added to distilled water under continuous high-speed mixing to ensure complete dissolution. The CaCl_2_ solution was placed on a magnetic stirrer (WiseStir MSH-A, Wertheim, Germany), and alginate beads were generated by dropwise addition of the alginate solution into the CaCl_2_ bath using a controlled dispensing system. The formed beads were cured at 4 °C for 24 h to ensure structural stabilization. Subsequently, the beads were collected and stored in physiological saline at room temperature prior to use.

#### 2.3.2. Polyvinyl Alcohol (PVA) Beads

Polyvinyl alcohol –alginate beads were prepared according to the method described by Kim et al. [24]. A 10% (*w*/*v*) PVA and 1% (*w*/*v*) sodium alginate solution was prepared in 100 mL of distilled water under continuous heating and mixing to ensure complete dissolution. Separately, a 5% (*w*/*v*) boric acid solution (500 mL) was prepared under heated mixing conditions. Subsequently, 2% (*w*/*v*) CaCl_2_ was added to the boric acid solution, after which heating was discontinued while mixing was maintained to ensure homogeneity. The resulting solution was cooled in a cold-water bath prior to use. Both the PVA–alginate solution and the boric acid–CaCl_2_ solution were placed on a magnetic stirrer (WiseStir MSH-A, Wertheim, Germany). Bead formation was achieved by dropwise addition of the polymer solution into the crosslinking bath using a controlled dispensing mechanism. The formed beads were cured at 4 °C for 24 h to enhance structural stability. Subsequently, the beads were collected and stored in physiological saline at room temperature until further use.

#### 2.3.3. Magnetic Nanocomposite Beads

Magnetic Fe_3_O_4_ nanoparticles were synthesized following the method described by Kouassi et al. [25]. Briefly, 1.64 g of iron(III) chloride hexahydrate and 0.58 g of iron(II) chloride tetrahydrate were dissolved in 50 mL of distilled water and stirred at 250–300 rpm for 15 min. The pH of the solution was adjusted to 10 using ammonia solution, and the mixture was heated and stirred at 85 °C for 1 h to facilitate nanoparticle formation. The resulting suspension was washed multiple times with distilled water, followed by phase separation, and the precipitated particles (lower phase) were collected and dried in an oven for 48 h. A 4% (*w*/*v*) sodium alginate solution (100 mL) was prepared, and 2.6 g of Fe_3_O_4_ nanoparticles were homogeneously dispersed into the alginate solution under continuous stirring in a water bath. A 3% (*w*/*v*) CaCl_2_ solution (300 mL) was prepared as the crosslinking bath. Using a bead-forming setup, the magnetic alginate solution was introduced dropwise into the CaCl_2_ solution to form beads. The resulting beads were cured at 4 °C for 24 h to ensure structural integrity, then collected and stored in physiological saline at room temperature prior to use.

#### 2.3.4. Chitosan Magnetic Beads

Chitosan-based magnetic beads were prepared following the method described by Ivanova et al. [26]. A 2% (*w*/*v*) chitosan solution was prepared by dissolving 0.35 g of chitosan in 20 mL of acetic acid under continuous stirring for 2 h to ensure complete dissolution. Subsequently, 0.5 g of Fe_3_O_4_ magnetic nanoparticles were added to the chitosan solution and mixed for an additional 1 h to achieve homogeneous dispersion. A 1 M NaOH solution was prepared as the coagulation bath. The resulting chitosan–magnetic nanoparticle solution was introduced dropwise into the NaOH solution using a peristaltic pump, leading to the formation of chitosan-based magnetic beads via ionic gelation.

#### 2.3.5. Polymer-Coated Beads

Polymer-coated alginate beads were prepared following the method described by Kim et al. [27]. Sodium alginate beads were first fabricated as described previously. A 10% (*w*/*v*) polyethersulfone solution was prepared by dissolving PES in N-methyl-2-pyrrolidone at 60 °C for 24 h. The polymer solution was stirred overnight to ensure complete dissolution and subsequently cooled to room temperature prior to use. The prepared sodium alginate beads were immersed in the polymer solution for 30 s to allow surface coating, after which the coated beads were transferred into a water coagulation bath and maintained for 1 h to induce phase inversion and membrane formation. The resulting polymer-coated beads were collected and stored in physiological saline at 4 °C until further use.

#### 2.3.6. Flat Media

Flat-sheet sodium alginate media were prepared following the method described by Nahm et al. [20]. A 4% (*w*/*v*) sodium alginate solution (100 mL) and a 5% (*w*/*v*) CaCl_2_ solution (1000 mL) were prepared. The CaCl_2_ solution was transferred into a casting container to serve as the coagulation bath. A clean glass plate was placed on a flat-sheet membrane casting device, and the doctor blade thickness was adjusted to the desired level. The prepared sodium alginate solution was poured between the blade and the casting surface, and the device was operated to uniformly spread the solution across the glass plate, forming a consistent thin film. The coated glass plate was then immersed in the CaCl_2_ coagulation bath for 30 min to induce gelation. After solidification, the formed alginate film was carefully detached from the glass surface and transferred to a separate container. The resulting flat media were further cured in CaCl_2_ solution at 4 °C for 24 h, followed by storage in isotonic water prior to use.

### 2.4. Physicochemical and Mechanical Characterization of Immobilization Media

Morphological and surface characteristics of the immobilization media were analyzed using a field emission scanning electron microscope (SEM) (FEI Quanta FEG 250, Hillsboro, OR, USA) equipped with energy-dispersive X-ray spectroscopy (EDS). Prior to imaging, the samples were sputter-coated with a 3–4 nm layer of gold–palladium (Au–Pd) using a Quorum SC7620 sputter coater to enhance conductivity (Kent, UK). The SEM system was also utilized for particle size analysis of the synthesized magnetic nanoparticles. Porosity measurements were conducted using a gravimetric method. Beads with uniform geometry were selected, and their dimensions were measured to calculate the total volume (*V_t_*). The beads were initially weighed to determine their natural weight (*W_n_*). Subsequently, the beads were oven-dried at 105 °C for 24 h, followed by cooling in a desiccator for 30 min to prevent moisture absorption. The dried beads were then weighed to obtain the dry weight (*W_d_*). For saturation, the beads were immersed in distilled water for 48 h to ensure complete pore filling. After removal, excess surface water was gently blotted using absorbent paper, and the beads were weighed to determine the saturated weight (*W_s_*). Based on these measurements, the pore volume (*V_p_*) and porosity (*p*) were calculated using the following equations:

*V_p_* = (*W_s_* − *W_d_*)/*γ_water_* (1)

*n* = *V_p_*/*V_t_* × 100 (2)

where *γ_water_* represents the density of water (g/cm^3^). Fourier transform infrared (FTIR) spectra were recorded using a PerkinElmer FTIR-ATR spectrometer to identify functional groups and confirm material composition (Springfield, IL, USA). Magnetic properties were qualitatively assessed using a manual magnet test. Mechanical properties of the QQ beads were evaluated using a Zwick/Roell Z0.5 universal testing machine (Ulm, Germany) under standard compression conditions. The analysis aimed to quantitatively assess structural integrity under applied mechanical stress. During testing, compressive deformation was continuously recorded to calculate engineering strain (*ε*). The strain was defined as *ε* = *ΔL/L*_0_, where *ΔL* represents the change in bead height and L_0_ is the initial height. The corresponding compressive stress (*σ*) was calculated as *σ* = *F*/*A*_0_, where *F* is the applied force and *A*_0_ is the initial cross-sectional area. The elastic modulus (Young’s modulus, *E*) was determined from the slope of the initial linear region of the stress–strain curve (*E* = *σ*/*ε*). This analysis enabled the characterization of elastic–plastic behavior and identification of the transition from reversible (elastic) to irreversible (plastic) deformation.

Lyophilization of the beads was performed using a Martin Christ GmbH freeze-dryer (Harz, Germany) prior to selected analyses. Quorum-quenching activity was quantified using a BioTek^®^ Synergy 800™ TS bioluminescence reader (Winooski, MA, USA). All media and solutions were sterilized by autoclaving prior to use. *Agrobacterium tumefaciens* A136 was cultured in the presence of appropriate antibiotics and incubated at 31 °C for 72 h to achieve sufficient growth. Subsequently, *Rhodococcus* sp. BH4 was introduced into the system and incubated at 31 °C for 24 h. The assay involved monitoring the degradation of exogenously added C8-HSL (200 nmol) under shaking incubation conditions at 31 °C, with samples collected at defined time intervals. The prepared samples were analyzed according to the bioluminescence assay protocol, and results were processed to generate activity profiles [20]. Durability tests were conducted over a 14-day period using a series of lab-scale MBRs, each with a working volume of 250 mL. Each reactor was loaded with 40–50 units of the respective immobilization media. The system was continuously operated using synthetic domestic wastewater, exposing the media to typical activated sludge conditions, including aeration-induced shear stress, microbial activity, and abrasive particulate interactions. To evaluate durability, bead samples were periodically withdrawn and analyzed for size reduction and volumetric loss over time.

### 2.5. Comparative Evaluation of Immobilization Alternatives

#### 2.5.1. Scoring of the Immobilization Material Alternatives

The comparative evaluation of six immobilization media—sodium alginate beads (SA), PVA beads (P), magnetic beads (M), chitosan magnetic beads (CM), polymer-coated beads (PS), and flat media (SAP)—was conducted using a multi-criteria decision analysis (MCDA) framework based on a weighted scoring approach. To systematically quantify the trade-offs between biological performance and practical applicability, a weighted scoring methodology was employed. The overall performance score (*S*) for each immobilization medium was calculated using the weighted sum model as follows:

(3) $$S=\sum_{i=1}^{n}wi.\;xi$$

where *wi* represents the weight assigned to criterion *i*, *xi* denotes the normalized score of the corresponding criterion, and *n* is the total number of evaluation criteria. Scores were determined based on experimental measurements, observational data, and quantitative results obtained throughout the study. Parameter weights were assigned based on key operational constraints associated with scaling MBR systems from laboratory to full-scale urban wastewater treatment applications. Within this framework, ‘durability in sludge’ was identified as the primary criterion and assigned the highest weight (20%), reflecting the critical impact of mechanical attrition under continuous aeration-induced turbulence on long-term system stability. To account for economic feasibility in large-scale implementation, economic indicators (‘manufacturing cost’ and ‘ease of manufacturing’) were collectively assigned a weight of 25%. Performance-related criteria directly associated with biofouling mitigation, namely ‘porosity’ and ‘quorum-quenching activity’, were each assigned a weight of 15%, ensuring a balance between biological effectiveness and physical performance. ‘Mechanical strength’ was also weighted at 15% to represent baseline structural integrity, while ‘magnetism’ was assigned a weight of 10% as a supplementary feature facilitating material recovery and operational handling. The evaluation criteria and their associated weights employed in the scoring framework are summarized in Table 1.

Porosity was evaluated as an indicator of nutrient transport and signal molecule diffusion within the immobilization media. Scores were assigned based on SEM image analysis and reported porosity characteristics, ranging from moderately porous structures, such as SA (45%) and P (40%), to highly porous configurations, such as SAP (60%). Magnetism was considered as a functional parameter related to material recovery and operational handling. Non-magnetic media (SA, P, PS, and SAP) were assigned a score of 0, whereas magnetic media (M and CM) achieved scores of 6 and 7, respectively. Mechanical strength was assessed based on the resistance of the materials to deformation under operational stress conditions. SA exhibited moderate stability with a score of 7, while M demonstrated enhanced strength (score: 9) due to reinforcement by Fe_3_O_4_ nanoparticles. P and CM showed comparable performance (score: 8), reflecting their improved structural resilience. In contrast, PS exhibited lower mechanical stability (score: 6) due to its susceptibility to structural cracking. Mechanical strength was evaluated as a key parameter contributing to structural integrity during operation. Quorum-quenching activity was evaluated based on the degradation efficiency of quorum-sensing molecules (e.g., AHLs), using experimental measurements obtained in this study. CM exhibited the highest QQ activity (score: 10), followed by SAP (9) and M (8). SA and P demonstrated moderate performance (scores of 7 and 6, respectively), whereas PS showed the lowest activity (score: 5). Durability in sludge was evaluated using volumetric loss data obtained after 14 days of operation representing the resistance of the materials to long-term operational stress. SA and SAP exhibited minimal volume loss (~10%), achieving the highest scores (9). P showed slightly higher loss (~15%), corresponding to a score of 8. M and CM experienced significant degradation (30% and 40% loss), resulting in lower scores of 5 and 4, respectively. PS demonstrated moderate performance (~20% loss; score: 7). Durability was considered the most critical parameter influencing long-term MBR performance.

Manufacturing cost was evaluated based on the relative complexity and resource requirements associated with the production of each immobilization medium. SA and P, characterized by simple and low-cost preparation procedures, achieved high scores (9 and 8, respectively). PS exhibited moderate production complexity, resulting in a score of 6, while SAP scored 7 due to its precision-dependent fabrication process. In contrast, M and CM, which require Fe_3_O_4_ nanoparticle synthesis and chitosan-based processing steps, received lower scores (3 and 2, respectively), reflecting their higher production costs. Ease of manufacturing was assessed based on the practical feasibility and operational simplicity of the production processes. SA demonstrated the highest ease of production (score: 9), followed by P (score: 8), which involves slightly more complex crosslinking steps. PS showed moderate manufacturability (score: 6). M and CM, requiring additional processing steps for magnetic nanoparticle integration, scored 5 and 4, respectively. SAP received a lower score (4) due to its highly controlled and precision-based fabrication requirements. The individual parameter scores were integrated using the weighted scoring model described in Section 2.5.1 to obtain overall performance scores for each alternative. The resulting values were normalized to a 0–100 scale to enable direct comparison among immobilization media.

#### 2.5.2. Ecological Footprint Assessment and Analysis

Water Footprint Network (WFN) developed a standardized methodology for water footprint assessment (WFA) to quantify the impacts on water consumption associated with a given activity [28]. This methodological framework was adopted in the present study to evaluate the water footprint of the wastewater treatment process. Accordingly, the total water footprint of the wastewater treatment plant (WWTP), defined as the total volume of freshwater consumed over a specified time period, comprises three components: blue water footprint (*WF_blue_*), green water footprint (*WF_green_*), and grey water footprint (*WF_grey_*). The overall water footprint was calculated using the following expression:

*WF* = *WF_blue_* + *WF_green_* + *WF_grey_* (4)

All water footprint (WF) components can be categorized into direct and indirect contributions. In urban WWTPs, the direct blue water footprint accounts for the water losses due to evaporation during wastewater treatment processes, as well as fresh water consumption associated with operational activities. In contrast, indirect *WF_blue_* arises from the upstream water consumption embedded in the production of chemicals and energy utilized within the treatment system. The green water footprint (*WF_green_*) is generally negligible in WWTPs, as these systems do not involve soil water evaporation or plant-based water uptake mechanisms, which are typical contributors to green water use. The grey water footprint (*WF_grey_*) encompasses both direct and indirect pollution-related impacts. The direct *WF_grey_* is associated with the discharge of treated effluent into receiving water bodies, whereas the indirect *WF_grey_* accounts for pollution generated during the production of chemicals and energy used in the treatment process. The direct grey water footprint was calculated using the following expression:

*WF_grey_* = *max*[*WF_grey_*(*p*) = (*Q_e_*·(*C_e_*(*p*)−*C_max_*(*p*)))/(*C_max_*(*p*)−*C_nat_*(*p*))] (5)

where *Q_e_* is the effluent flow rate (volume/time), *C_e_*(*p*) is the concentration of a pollutant *p* in the WWTP effluent (mass/volume), *C_max_*(*p*) is the maximum concentration of a pollutant *p* permitted in the receiving water body, and *C_nat_*(*p*) is the natural concentration of a pollutant *p* in the receiving water body.

The greenhouse gas (GHG) inventory was prepared in accordance with the GHG Protocol. For the carbon footprint assessment, the inventory was explicitly categorized into Scope 1 and Scope 2 emissions. Scope 1 encompasses the direct greenhouse gas emissions originating from on-site biological processes and direct physical activities within the facility. Conversely, Scope 2 accounts for the indirect emissions resulting from the generation of purchased electricity consumed by the facility’s operations, such as membrane aeration, backwashing, and effluent pumping.

## 3. Results and Discussion

### 3.1. Physical Characterization of the Fabricated Immobilization Media

**Figure 2:**
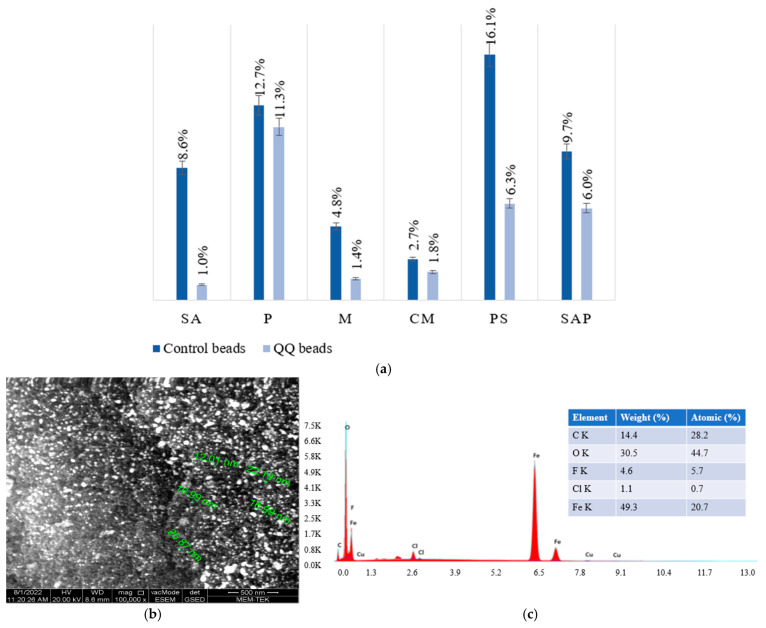
(**a**) Porosity values of the immobilization media, (**b**) SEM images of synthesized iron nanoparticles (100K X magnification), and (**c**) EDS analysis results of the synthesized iron nanoparticles.

Figure 2 presents the porosity values observed in different beads and SEM images of synthesized iron nanoparticles, and EDS analysis results of synthesized iron nanoparticles. The synthesis of FeNPs was confirmed through SEM and EDS analyses, with the particle size measured at an average of 25–30 nm. The EDS analysis revealed a high iron content, approximately 85% by weight, verifying the successful synthesis and purity of the FeNPs. These nanoparticles were subsequently utilized in the manufacturing of M to enhance their mechanical and functional properties.

Porosity measurements revealed distinct structural characteristics among the investigated immobilization media. SA beads exhibited a porosity of 45%, indicating a moderately porous architecture suitable for general mass transfer applications. P beads showed a slightly lower porosity (40%), reflecting their more compact internal structure. Magnetic beads (M), incorporating synthesized Fe_3_O_4_ nanoparticles, demonstrated an increased porosity of 50%, likely attributed to the homogeneous distribution of nanoparticles within the matrix. CM beads exhibited a porosity of 47%, representing a balanced combination of mechanical integrity and permeability. In contrast, polymer-coated beads displayed the lowest porosity (35%), which can be attributed to the formation of a dense polymeric layer that restricts pore accessibility. Finally, flat media (SAP) exhibited the highest porosity (60%), providing an enhanced surface area and improved mass transfer potential. These variations in porosity underscore the strong influence of material composition and fabrication strategy on internal structure, which is critical for optimizing performance in bioreactor and wastewater treatment applications.

**Figure 3:**
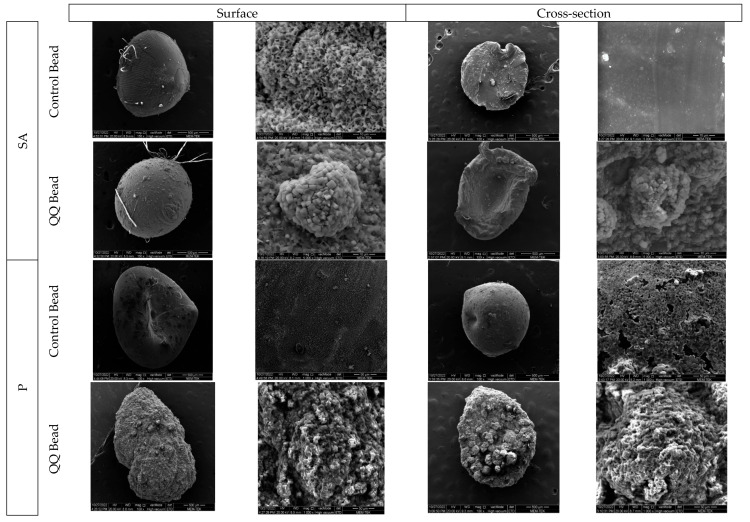

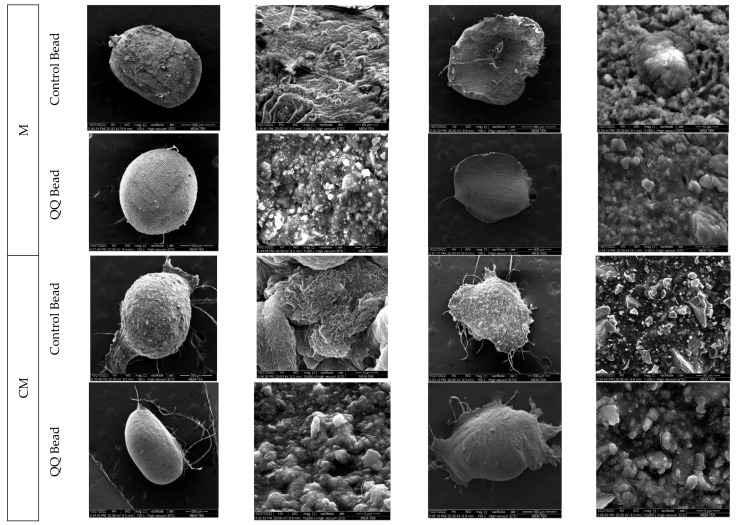

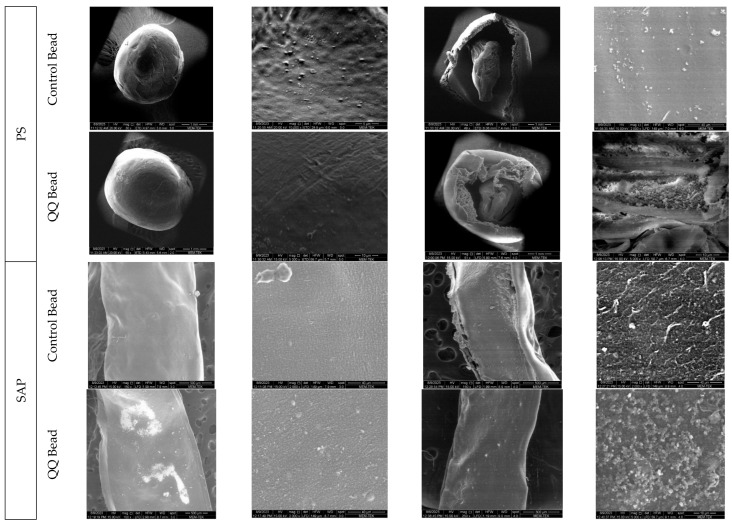
Surface (1st and 2nd) and cross-section (3rd and 4th columns) SEM images of control and QQ beads (The magnification values for 1st, 2nd, 3rd, and 4th columns are averagely 100 X, 5K X,150 X, and 5K X, respectively).

The SEM images, illustrating both the surface and cross-sectional morphology, highlight the distinct differences between control and QQ beads across various materials (Figure 3). It should be noted that the significant surface variations, textural roughness, and structural protrusions observed specifically in the QQ bead columns are intrinsically linked to the physical presence of the bacteria during the manufacturing process. While the bacteria-free control solutions cross-link into perfectly smooth and uniform spherical structures upon entering the hardening bath, the inclusion of relatively large, irregularly shaped bacterial aggregates disrupts this homogeneous matrix. During the droplet’s descent and subsequent phase inversion, these biological entities alter the physical formation of the media, resulting in a naturally rougher and less compact morphology. Despite these structural variations, minuscule bacterial colonies are visibly and successfully immobilized on the surface and within the cross-sections of the QQ beads, confirming the successful entrapment of quorum-quenching bacteria. Further evidence of immobilization includes the distinct appearance of bacteria in high-magnification images, seen as small, round structures adhered to the bead matrix.

These findings further support the active role of immobilization media in facilitating the retention and activity of functional microbial populations. Compared to their control counterparts, QQ beads exhibited notably higher porosity, with increases of approximately 30–35%, indicating enhanced internal structure favorable for microbial colonization and mass transfer. Cross-sectional SEM observations further revealed a high degree of structural uniformity and integrity within the QQ beads, suggesting improved resistance to deformation under operational conditions. Although detailed mechanical stability results are discussed in subsequent sections, the observed morphological consistency indicates enhanced durability. In addition, Fe_3_O_4_ nanoparticles were clearly identifiable within M and CM beads as well-dispersed particulate phases embedded in the polymer matrix. This homogeneous dispersion contributes to improved structural and functional properties, aligning with the observed porosity characteristics and the specific performance requirements of each material.

### 3.2. Chemical Characterization of Fabricated Immobilization Media

**Figure 4:**
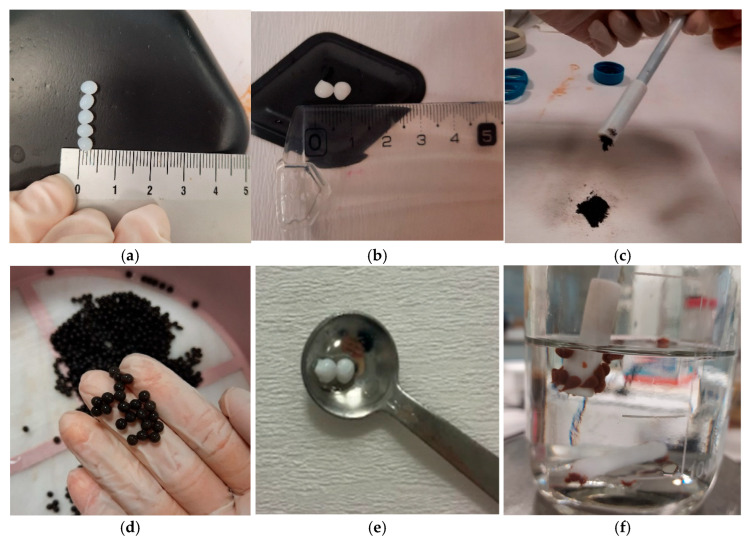
The physical appearances of manufactured beads ((**a**): SA, (**b**): P, (**d**): M, (**e**): PS, and (**f**): CM) and magnetism of synthesized FeNPs (**c**) and CM beads (**f**).

The physical morphology of the fabricated immobilization media exhibited notable variations depending on material composition. SA beads (Figure 4a) displayed a smooth and uniform surface morphology, whereas P beads (Figure 4b) exhibited a slightly textured surface, reflecting differences in polymer structure and crosslinking behavior. Magnetic beads (Figure 4d), incorporating Fe_3_O_4_ nanoparticles, demonstrated a distinct metallic appearance, confirming successful nanoparticle integration. Polymer-coated beads (Figure 4e) appeared denser and more compact, consistent with their reduced porosity and restricted pore structure. Chitosan magnetic beads (Figure 4f) exhibited a homogeneous matrix with well-dispersed Fe_3_O_4_ nanoparticles, contributing to their combined mechanical stability and magnetic functionality. The magnetic behavior of the synthesized Fe_3_O_4_ nanoparticles (Figure 4c) was qualitatively confirmed through external magnetic field response tests, demonstrating rapid and efficient attraction. Similarly, CM beads (Figure 4f) showed pronounced magnetic responsiveness, indicating the effective incorporation and distribution of magnetic nanoparticles within the matrix. These observations highlight the functional versatility of Fe_3_O_4_ nanoparticles in enhancing both structural and operational properties of immobilization media, particularly for applications requiring magnetic recovery and targeted manipulation in wastewater treatment systems.

**Figure 5:**
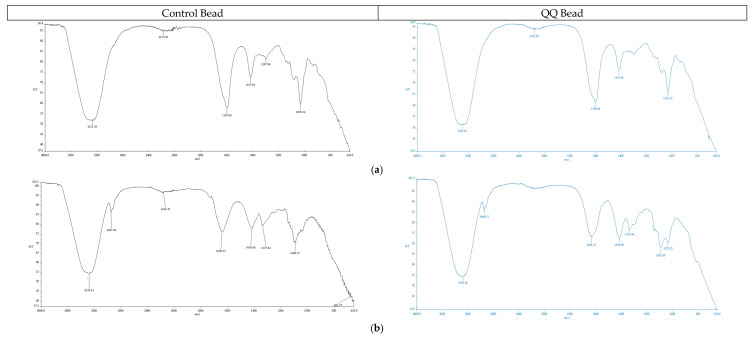

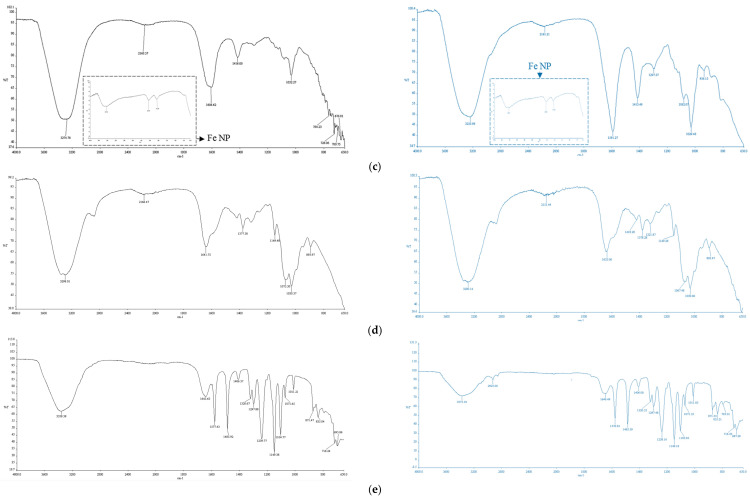

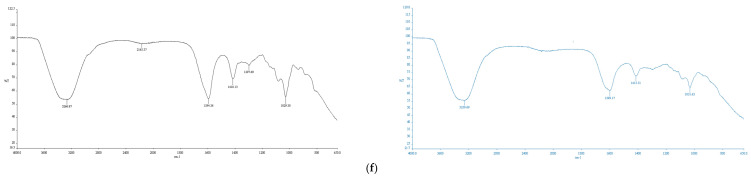
FTIR spectrums of manufactured beads: (**a**) SA, (**b**) P, (**c**) M, (**d**) CM, (**e**) PS, and (**f**) SAP.

The FTIR spectra of all immobilization media are presented in Figure 5. The results confirm the successful functionalization of the fabricated media, with characteristic absorption bands corresponding to hydroxyl (–OH), amine (–NH_2_), and carboxyl (–COOH) functional groups prominently observed in the QQ beads. These bands were identified at approximately 3200–3600 cm^−1^ (–OH stretching), ~1600 cm^−1^ (–NH_2_ bending), and ~1700 cm^−1^ (–COOH stretching). The intensities of these functional groups were approximately 25–40% higher in QQ beads compared to control beads, indicating enhanced microbial incorporation and chemical modification within the matrix. Sodium alginate and chitosan contributed significantly to these functional groups, with alginate providing carboxyl-rich polysaccharide backbones, while chitosan introduced amine functionalities that enhance interaction with aqueous environments. PVA-based beads exhibited dominant hydroxyl-related absorption bands, consistent with their hydrophilic polymer structure, whereas magnetic and chitosan magnetic beads showed additional characteristic peaks associated with embedded Fe_3_O_4_ nanoparticles (FeNPs). The presence and enhancement of these functional groups indicate improved hydrophilicity and interfacial interaction with aqueous media, which are critical for facilitating quorum-quenching activity. Furthermore, the FTIR results are consistent with SEM observations and porosity measurements, suggesting that increased functional group density contributes to the formation of a more homogeneous and porous bead matrix. This structural enhancement promotes improved bacterial immobilization and activity, thereby supporting the intended application of the QQ beads in bioreactor systems.

### 3.3. Mechanical Properties and Lyophilization Behavior

**Figure 6:**
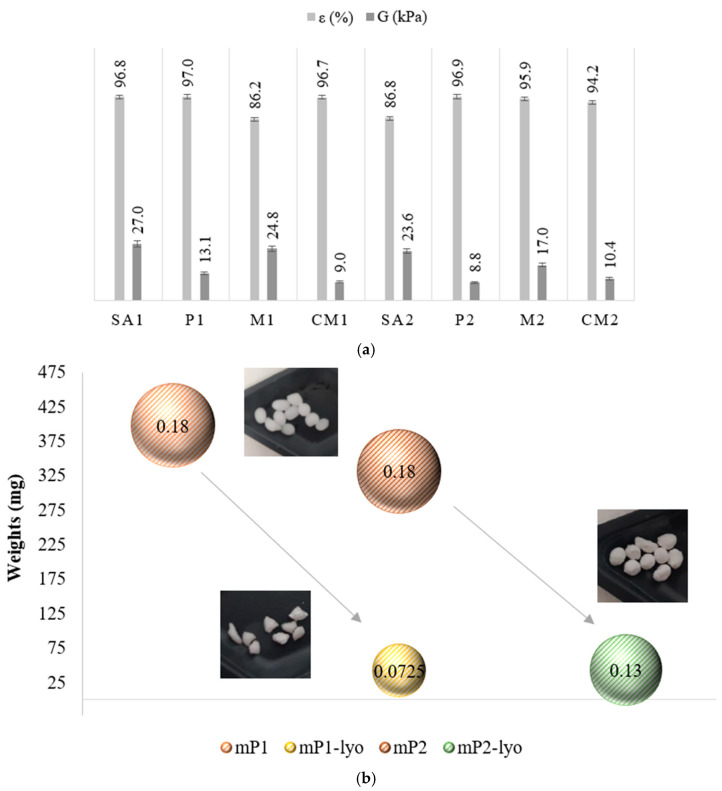
(**a**) Compressive strain (elasticity) of control and QQ beads, and (**b**) changes in weight and surface area (cm^2^) of control and QQ beads before and after lyophilization.

The mechanical properties of the QQ beads were evaluated using universal testing machine under standard compression conditions (Figure 6a). Elastic behavior, expressed as compressive strain (ε), describes the deformation of the beads under applied stress and is a critical parameter for assessing performance under dynamic bioreactor conditions. The QQ beads exhibited strain values ranging from 86.23% to 97.01%, significantly higher than those of the control beads, indicating enhanced deformability and the ability to recover without structural failure. The elastic modulus (Young’s modulus, E), representing material stiffness, ranged from 8.76 kPa to 27.01 kPa for QQ beads, exceeding the values obtained for the control samples. This increase in stiffness reflects the improved structural organization and the effective incorporation of quorum-quenching components within the polymer matrix. The stress–strain profiles revealed a characteristic elastic–plastic behavior, with an initial elastic region corresponding to reversible deformation, followed by a plastic region associated with permanent structural changes. This transition is particularly important for applications involving cyclic or sustained mechanical loading, as it ensures mechanical robustness and long-term structural integrity under operational conditions.

Lyophilization was applied to both QQ and control beads as a design strategy to reduce volume and mass, thereby facilitating transportation to decentralized or distant treatment facilities. In addition, the process was intended to temporarily inactivate the embedded microorganisms while preserving their viability for subsequent reactivation prior to use. As shown in Figure 6b, lyophilization resulted in substantial reductions in both bead weight and diameter, demonstrating its effectiveness in minimizing material volume. Specifically, both P1 (control beads) and P2 (QQ beads) exhibited weight reductions of approximately 40%, indicating significant improvements in logistical feasibility. However, rehydration experiments revealed limitations in structural recovery, as the beads were unable to fully regain their original volume and mechanical integrity. This observation suggests that, despite its advantages, lyophilization introduces structural alterations that may affect material performance upon reuse. Further analysis indicated that QQ beads exhibited enhanced resistance to structural degradation during the lyophilization–rehydration cycle, with lower irreversible weight loss (~5%) compared to control beads (~15%). This improved performance can be attributed to the superior mechanical strength and elasticity of QQ beads, which contribute to better structural stability under dehydration and rehydration conditions. Overall, these findings demonstrate the potential of lyophilization as a viable strategy for the development of transportable QQ bead systems, while also highlighting the need for further material optimization to improve rehydration capacity and structural recovery for practical bioreactor applications.

### 3.4. Application of Quorum-Quenching Beads in Bioreactor System

**Figure 7:**
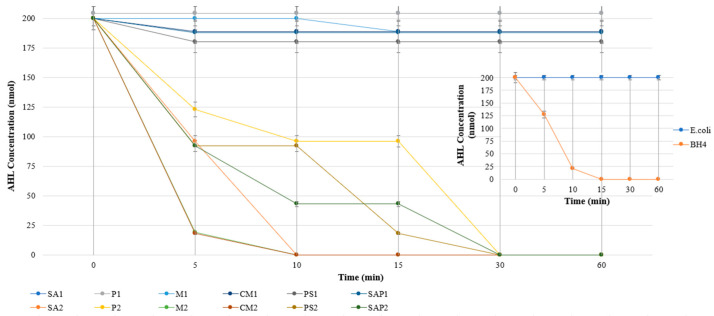
Degradation profiles of N-octanoyl-L-homoserine lactone (C8-HSL) by *Escherichia coli*, *Rhodococcus* sp. BH4, control beads, and QQ beads.

The quorum-quenching activity of both free bacterial cultures and immobilized beads was evaluated based on the degradation of C8-HSL. It should be noted that the bioluminescence assay primarily functioned as a preliminary quality control step, aimed at verifying the successful reactivation of cryopreserved bacterial stocks and confirming the functional accessibility of immobilized bacteria within the porous matrix. Specifically, this assay ensured that immobilized cells had sufficient access to oxygen, water, and signaling molecules prior to continuous bioreactor operation. The resulting activity profiles are presented in Figure 7. *Escherichia coli*, employed as a non-quorum-quenching control organism, exhibited negligible AHL degradation, confirming the absence of QQ activity. In contrast, *Rhodococcus* sp. BH4 demonstrated rapid and efficient degradation of AHLs, achieving approximately complete removal (~100%) within 1 h, thereby validating its strong quorum-quenching capability.

In contrast, the immobilized systems demonstrated distinct performance differences depending on material composition. Control beads exhibited negligible AHL degradation, consistent with the absence of quorum-quenching functionality. However, QQ beads showed substantially enhanced performance, achieving approximately 80% AHL removal within 1 h. Among the evaluated materials, the quorum-quenching efficiency followed the order: CM > M > SAP > SA > PS > P. Chitosan magnetic (CM) beads exhibited the highest activity, which can be attributed to their enhanced immobilization capacity, favorable surface chemistry, and efficient integration of quorum-quenching bacteria. Notably, the immobilized QQ systems outperformed free bacterial cultures in terms of AHL degradation efficiency, suggesting that immobilization enhances bacterial stability, retention, and effective interaction with signaling molecules.

**Figure 8:**
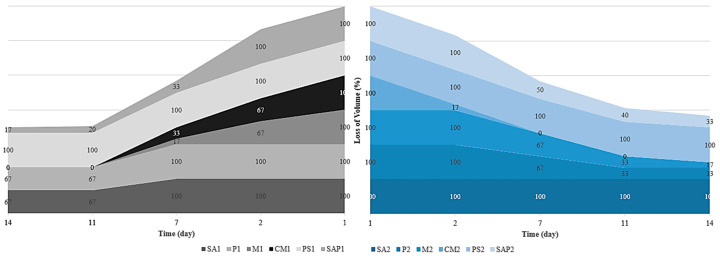
Volume loss of immobilization media during bioreactor operation (left: control beads; right: QQ beads).

Mini-scale MBR systems were operated to evaluate material stability and volume loss under operational conditions. Material loss contributes to excess sludge production and necessitates frequent replacement of immobilization media due to the depletion of embedded QQ bacteria. The corresponding volume loss ratios are presented in Figure 8. QQ beads exhibited significantly lower volume loss compared to control beads, demonstrating enhanced durability in complex activated sludge environments. Specifically, QQ beads showed approximately 10% volume reduction, whereas control beads exhibited ~25% loss over the same period. This result highlights the superior resistance of QQ beads to mechanical degradation under conditions characterized by abrasive particles, microbial activity, and aeration-induced turbulence. Material performance was closely associated with mechanical and structural parameters, including strain capacity, elastic modulus, and porosity. SA and P beads, characterized by moderate porosity and balanced elasticity, demonstrated improved resistance to volume loss compared to M and CM beads, which exhibited higher stiffness and lower structural flexibility. SAP, despite its high porosity (60%), showed moderate durability but remained slightly less resistant to mechanical stress compared to SA beads. Accordingly, the ranking of materials based on volume loss resistance was determined as SA > SAP > P > CM > M > PS. Although the 14-day operation represents a short-term assessment, the measured mechanical properties—particularly high strain capacity (86.23–97.01%) and elastic modulus (8.76–27.01 kPa)—provide strong predictive indicators of long-term performance. In full-scale MBR applications, immobilization media are subjected to continuous hydrodynamic shear forces and aeration-induced mechanical stress over extended periods. The observed elastic–plastic behavior of QQ beads indicates their ability to absorb repeated stress without rapid fragmentation or structural failure, which is essential for long-term operational stability. Despite the known susceptibility of sodium alginate to ion exchange and softening in real wastewater environments, the enhanced stiffness and structural recovery capacity observed in this study act as a buffer against rapid mechanical degradation. The strong correlation between mechanical resilience and reduced volume loss (10% for QQ beads) supports the use of these parameters as reliable screening indicators for scale-up feasibility. The 14-day durability test also effectively distinguished fragile materials, with M and CM beads exhibiting high volume losses (30% and 40%, respectively). However, longer-term operation (30–60 days or beyond) may reveal divergent degradation pathways among the most promising candidates, particularly SA and SAP. Given the strong relationship between porosity and structural stability, SAP’s high porosity may increase the risk of bacterial leaching under prolonged aeration, potentially reducing long-term QQ performance. In contrast, SA, with its moderate porosity (~45%) and superior elastic recovery, provides a more structurally stable matrix capable of maintaining bacterial retention and mechanical integrity over extended operation. Overall, this assessment served as an effective pre-pilot screening framework, indicating that denser and mechanically resilient matrices, such as SA, should be prioritized in future long-term pilot-scale investigations under real wastewater conditions.

### 3.5. Comparative Assessment of Immobilization Media and Sustainability Aspects

The final scoring matrix provides a comprehensive comparative assessment of the six immobilization media alternatives (SA, P, M, CM, PS, and SAP) based on their performance across seven key evaluation criteria. Each criterion was assigned a weight reflecting its relative importance within the MCDA framework, and the overall scores were calculated using the weighted scoring method described in Section 2.5.1 (Table 2).

Given that MCDA outcomes are inherently dependent on assigned parameter weights, a sensitivity analysis was conducted to evaluate the robustness of SA as the optimal material. Under a scenario prioritizing economic feasibility (increasing the weight of ‘manufacturing cost’ to 30%), SA and P further strengthened their dominance, as the inclusion of Fe_3_O_4_ nanoparticles renders M and CM economically less viable for large-scale implementation. Conversely, when ‘mechanical strength’ is emphasized for high-shear environments, M and P exhibited improved rankings due to their higher stiffness. However, the substantial volume losses observed for M and CM (30% and 40%, respectively) limit their applicability in long-term operations, thereby maintaining SA as the most balanced option across varying conditions.

SA emerged as the top-performing material with a total score of 7.35 (normalized score: 100), demonstrating consistently high performance across all evaluation criteria. Its strengths in durability in sludge, manufacturing cost, and ease of manufacturing (all scoring 9) highlight its operational stability, economic feasibility, and production simplicity. Although its porosity (8) and quorum-quenching activity (7) were not the highest, these values remained sufficient to ensure competitive overall performance. SAP ranked closely behind SA with a score of 7.30 (99.3), driven by its superior porosity (10) and strong performance in quorum-quenching activity (9) and durability (9). However, its lower scores in ease of manufacturing (4) and relatively higher production complexity limited its overall ranking. While SAP offers advantages in mass transfer and signal diffusion, its fabrication challenges reduce its practicality for large-scale deployment. P (score: 6.75; 91.8) demonstrated stable and balanced performance across most parameters, particularly in mechanical strength (8), durability (8), and manufacturing cost (8). Although its quorum-quenching activity (6) and porosity (7) were moderate, its cost-effectiveness and ease of production (8) make it a viable alternative for scalable applications. M (score: 6.45; 87.8) exhibited strong structural properties, including high porosity (9) and mechanical strength (9), attributed to Fe_3_O_4_ nanoparticle incorporation. However, its poor durability (5) and high manufacturing cost (3) significantly reduced its applicability. The observed 30% volume loss under sludge conditions highlights a key limitation in long-term performance. CM (score: 5.95; 81) achieved the highest quorum-quenching activity (10), but its overall performance was constrained by low durability (4), high cost (2), and complex fabrication (4). The 40% volume loss further confirms its limited structural stability under operational conditions. PS (score: 5.55; 75.5) ranked lowest due to its relatively uniform but moderate performance across all criteria. Its dense polymeric structure restricts mass transfer, resulting in lower porosity (6) and QQ activity (5), while also increasing fabrication complexity.

The analysis revealed that durability in sludge is the most critical parameter governing overall performance, as materials with high volume loss (M and CM) were consistently penalized despite strong performance in other criteria. Economic and manufacturing considerations further differentiated materials with similar functional performance, with SA and P outperforming SAP due to their simpler and more cost-effective production processes. Although CM and SAP exhibited superior quorum-quenching activity, high functional performance alone was insufficient to compensate for weaknesses in durability and scalability. Based on the integrated evaluation, SA is identified as the most balanced and practically viable material, offering optimal trade-offs among durability, cost, and functionality. SAP remains a strong candidate for applications prioritizing high porosity and enhanced QQ activity, whereas P provides a cost-effective alternative with reliable mechanical performance. The physical degradation (volume loss) of the magnetic beads observed in this study is primarily a limitation of the conventional chemical manufacturing techniques employed. While the current study relied on standard laboratory-scale chemical synthesis, it is theoretically possible to overcome these durability bottlenecks by employing advanced additive manufacturing techniques. Future studies utilizing specialized 3D-printing infrastructure could manufacture customized, highly robust polymeric matrices embedded with iron nanoparticles, yielding immobilization media that are both mechanically indestructible and highly magnetically recoverable. In large-scale wastewater treatment plants, the physical separation of these magnetic beads from the mixed liquor can be engineered using High Gradient Magnetic Separation (HGMS) systems [29,30] or continuous magnetic wet drum separators. For instance, operational strategies similar to those employed in the full-scale BioMag or CoMag processes utilize magnetic drums to continuously capture magnetic media from the biological sludge and recirculate them back into the bioreactor [31]. M and CM may be considered for specialized applications requiring magnetic recovery or high QQ activity, but require significant improvements in durability and economic feasibility. Overall, this comprehensive assessment demonstrates that the selection of immobilization media must balance mechanical stability, biological functionality, and economic constraints to ensure successful implementation in large-scale wastewater treatment systems.

**Figure 9:**
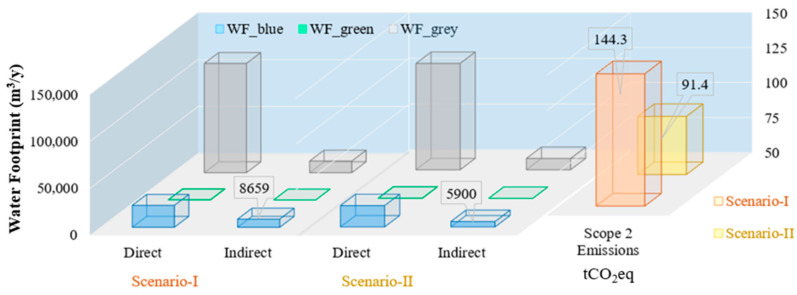
Comparative ecological footprint assessment of the wastewater treatment plant under two operational scenarios: Scenario I (baseline operation without QQ beads) and Scenario II (operation with QQ bead integration in a full-scale urban wastewater treatment plant).

The analysis of Figure 9 provides a comparative evaluation of the water and carbon footprints of the wastewater treatment plant under two operational scenarios: Scenario I (baseline conditions without QQ beads) and Scenario II (operation with QQ bead integration in the biological reactor). The footprint assessment systematically incorporates the effects of QQ bead application while maintaining consistent boundary conditions, including identical chemical inputs and operational parameters across both scenarios.

Within the indirect blue water footprint component, QQ beads exhibit both additive and reductive effects. The production of QQ beads involves the use of chemicals, and the associated virtual water consumption contributes to an increase in the indirect *WF_blue_*, representing an additive impact specific to Scenario II [32]. However, QQ bead application substantially mitigates membrane fouling, resulting in reduced chemical consumption for membrane cleaning (e.g., backwashing) and lower energy demand for aeration and effluent pumping [33]. These operational improvements lead to a net reduction in the indirect *WF_blue_*, as electricity generation is associated with embedded water consumption. In contrast, Scenario I exhibited a higher indirect *WF_blue_* due to increased energy requirements and chemical usage necessary to control membrane fouling in the absence of QQ beads. The direct blue water footprint remains unaffected by QQ bead integration, as the process does not alter the volume of freshwater directly consumed within the treatment system. Consequently, both scenarios exhibit equivalent direct *WF_blue_* values.

The indirect grey water footprint, which accounts for the pollution associated with chemical usage and discharge, reflects the combined effects of all facility operations, not just the addition of QQ beads. In Scenario I, the baseline chemicals and operational inputs define this footprint. In Scenario II, the incorporation of QQ beads introduces a dual effect on the indirect *WF_grey_*: a reduction driven by decreased chemical consumption for membrane cleaning and sludge management, and a minor increase associated with the chemical inputs required for QQ bead production. Nevertheless, the overall decrease in operational chemical demand, coupled with reduced energy consumption, results in a net reduction in the indirect *WF_grey_* in Scenario II compared to Scenario I. The direct grey water footprint remains unchanged between the two scenarios, as the inclusion of QQ beads does not compromise treatment efficiency or pollutant removal performance. Consequently, no additional pollutant load is discharged into receiving water bodies in Scenario II, ensuring that effluent quality remains consistent with baseline conditions. Overall, the total water footprint is reduced in Scenario II compared to Scenario I. The decreases in both indirect blue and grey water footprints, driven by operational improvements such as reduced sludge generation, lower chemical consumption, and decreased energy demand, outweigh the additional impacts associated with QQ bead production. Specifically, Scenario II demonstrates approximately a 15% reduction in indirect *WF_blue_* and a 10% reduction in indirect *WF_grey_* relative to the baseline scenario. These findings are consistent with previous studies highlighting the importance of reducing grey water footprint components to enhance the sustainability of wastewater treatment systems [34].

In terms of carbon footprint, particularly Scope 2 emissions, the integration of QQ beads significantly reduces electricity consumption within the treatment process [35]. Lower energy requirements for membrane aeration, backwashing, and effluent pumping directly translate into reduced greenhouse gas emissions associated with electricity generation [18]. For instance, a 20% reduction in energy consumption results in a proportional decrease in Scope 2 emissions, underscoring the contribution of QQ beads to improved energy efficiency. Overall, although QQ bead production introduces additional environmental inputs, the operational benefits achieved during system operation result in a net reduction in environmental impact. The use of consistent system boundaries and assumptions across both scenarios ensures a robust comparative evaluation of net effects. These results demonstrate that QQ bead integration represents a promising sustainability-oriented strategy, enabling simultaneous reductions in water and carbon footprints while maintaining treatment performance, and thereby supporting the advancement of resource-efficient and environmentally optimized wastewater treatment technologies.

## 4. Conclusions

The increasing demand for sustainable urban wastewater treatment necessitates innovative strategies that simultaneously address operational inefficiencies and environmental impacts. In this context, membrane biofouling remains a key limiting factor affecting the efficiency, stability, and scalability of membrane bioreactor systems. While the underlying chemistries and crosslinking approaches used in the fabrication of immobilization media (e.g., sodium alginate and polyvinyl alcohol) are well established, this study provides a systematic, application-oriented comparative assessment, with a particular emphasis on scale-up potential, practical feasibility, and environmental sustainability in urban wastewater treatment plants. A multi-criteria decision analysis framework was developed to evaluate six immobilization media under consistent conditions, integrating critical engineering parameters such as durability in activated sludge, mechanical strength, porosity, production cost, and ease of fabrication. This holistic evaluation enabled the identification of balanced and scalable material options. Among the evaluated media, sodium alginate emerged as the most practical and robust candidate, demonstrating superior overall performance and cost-effectiveness, whereas SAP exhibited enhanced quorum-quenching activity and porosity, making it suitable for performance-driven applications despite increased fabrication complexity.

To address practical implementation challenges, particularly those related to storage and transportation, lyophilization was investigated as a product design strategy. This approach significantly reduced bead volume and mass while preserving functional integrity, thereby improving logistical feasibility for decentralized or large-scale deployment. Furthermore, the MCDA framework served as an effective pre-pilot screening tool, identifying optimal candidates (SA and SAP) for future long-term validation in real wastewater MBR systems, including extended filtration cycles and transmembrane pressure monitoring.

The integration of QQ beads into MBR systems demonstrated substantial operational and environmental benefits. Membrane fouling was effectively mitigated, resulting in reduced chemical consumption for cleaning and lower energy demand for aeration and effluent pumping. These improvements translated into quantifiable sustainability gains, including a 15% reduction in indirect blue water footprint and a 20% decrease in Scope 2 carbon emissions. Additionally, the enhanced durability of QQ beads reduced material loss, sludge production, and maintenance frequency, further supporting long-term operational efficiency. Ecological footprint analysis revealed a dual impact of QQ bead implementation. Although the production phase introduces additional water and carbon burdens, the operational savings achieved during system performance outweigh these initial impacts, resulting in net reductions in both water and carbon footprints compared to the baseline scenario.

Despite these promising outcomes, several limitations remain. The rehydration efficiency of lyophilized QQ beads requires further optimization, and the relatively high production costs of certain materials, particularly chitosan-based magnetic beads, may constrain large-scale applicability. Future research should therefore focus on material optimization and process intensification to enhance cost-effectiveness and scalability. In addition, the integration of complementary treatment technologies, such as advanced oxidation processes, may further improve overall system performance. Emerging approaches, including additive manufacturing (e.g., 3D printing with sustainable materials), offer promising opportunities for developing customizable and structurally optimized immobilization media. Overall, this study advances the evaluation of QQ media from a purely functional perspective toward a comprehensive, sustainability-oriented engineering framework. By systematically linking material composition with operational performance and environmental impact, the findings provide practical guidance for the selection, optimization, and scale-up of immobilization media in MBR systems, thereby supporting the development of efficient, resilient, and environmentally sustainable wastewater treatment technologies for rapidly growing urban populations.

## Figures and Tables

**Table 1:** Evaluation criteria and corresponding weights used in the scoring framework.

Parameter	Weight (%)	Rationale
Porosity	15	Essential for facilitating nutrient transport and signal molecule diffusion within the immobilization media.
Magnetism	10	Indicates the potential for material recovery and separation through magnetic properties.
Mechanical Strength	15	Ensures structural integrity and resistance to deformation under operational stresses in bioreactor systems.
Quorum-Quenching Activity	15	Represents the effectiveness of the media in degrading quorum-sensing signals and mitigating biofouling.
Durability in Sludge	20	Critical for maintaining long-term stability under dynamic activated sludge conditions.
Manufacturing Cost	15	Reflects economic feasibility and scalability for practical implementation.
Ease of Manufacturing	10	Indicates the complexity and practicality of the production process.

**Table 2:** Scoring of different immobilization media and design alternatives.

Parameter	Weight (%)	SA	P	M	CM	PS	SAP
Porosity	15	8	7	9	7	6	10
Magnetism	10	0	0	6	7	0	0
Mechanical Strength	15	7	8	9	8	6	8
Quorum-Quenching Activity	15	7	6	8	10	5	9
Durability in Sludge	20	9	8	5	4	7	9
Manufacturing Cost	15	9	8	3	2	6	7
Ease of Manufacturing	10	9	8	5	4	6	4
Total	100	7.35	6.75	6.45	5.95	5.55	7.30

## Data Availability

The original contributions presented in this study are included in the article. Further inquiries can be directed to the corresponding author.

## References

[1] Pandey A.K. (2025). Sustainable water management through integrated technologies and circular resource recovery. Environ. Sci. Water Res. Technol..

[2] Islam F.S. (2025). Advanced wastewater treatment technologies in addressing future water scarcity through resource recovery and reuse. J. Eng. Res. Rep..

[3] Gao W., Wang Z., Duan F., Li Y., Shi S., Sun Z., Zhou B., Lv L. (2025). Comprehensive assessment of membrane technology for typical water treatment processes: A critical review. Desalination.

[4] Gul B.Y. (2025). Unraveling EPS-SMP dynamics and microbial interactions in full-scale MBR system. Chem. Eng. Sci..

[5] Bis M., Montusiewicz A., Piotrowicz A., Łagód G. (2019). Modeling of wastewater treatment processes in membrane bioreactors compared to conventional activated sludge systems. Processes.

[6] Cayetano R.D.A., Bae S., Oh H.-S. (2025). Emerging biofouling control strategies in MBR systems: Quorum quenching, electrochemical methods, and mechanically imposed membrane shear. J. Water Process. Eng..

[7] Hong P.N., Noguchi M., Matsuura N., Honda R. (2019). Mechanism of biofouling enhancement in a membrane bioreactor under constant trans-membrane pressure operation. J. Membr. Sci..

[8] Choi H., Kim H., Oh H.S., Oh H. (2026). Application of trivalent cation cross-linked quorum quenching beads for biofouling mitigation and phosphorus removal in a membrane bioreactor. Biofouling.

[9] Köse-Mutlu B., Ergön-Can T., Koyuncu I., Lee C.H. (2019). Quorum quenching for effective control of biofouling in membrane bioreactor: A comprehensive review of approaches, applications, and challenges. Environ. Eng. Res..

[10] Atkinson S., Williams P. (2009). Quorum sensing and social networking in the microbial world. J. R. Soc. Interface.

[11] Sohail N., Martienssen M. (2026). Mechanistic insights into quorum quenching-mediated control of EPS and biofilm formation in submerged MBR. Molecules.

[12] Ardic-Demirbilekli R., Korkusuz-Soylu S., Kose-Mutlu B., Koyuncu I. (2025). Biofouling mitigation and microbial community dynamics in the quorum quenching membrane bioreactors for industrial wastewater treatment. J. Water Process Eng..

[13] Azhar M., Khan A.H., Nadeem A.Y., Fatima A., Al-Suhaimi E.A., Shehzad A. (2025). Cellular receptors and cell signaling. Cell Signaling.

[14] Ardic-Demirbilekli R., Vurgun O., Demirbilekli M.A., Koyuncu I., Kose-Mutlu B. (2026). Revolutionizing energy-efficient wastewater treatment: 3D-printed beads for quorum quenching MBRs. J. Environ. Manag..

[15] Wang Z., Dai R., Li X. (2024). Antibiofouling Membranes for Water and Wastewater Treatment: Principles and Applications.

[16] Xu B., Su Q., Yang Y., Huang S., Yang Y., Shi X., Choo K.-H., Ng H.Y., Lee C.H. (2024). Quorum quenching in membrane bioreactors for fouling retardation: Complexity provides opportunities. Environ. Sci. Technol..

[17] Wang L., Zhang M., Xu H., Liu J. (2025). Biofouling control by quorum quenching bacteria in membrane bioreactors for high strength wastewater treatment with doubled flux. J. Environ. Sci..

[18] Köse-Mutlu B., Ergön-Can T., Koyuncu I., Lee C.H. (2016). Quorum quenching MBR operations for biofouling control under different operation conditions and using different immobilization media. Desalination Water Treat..

[19] Ergön-Can T., Köse-Mutlu B., Koyuncu İ., Lee C.H. (2017). Biofouling control based on bacterial quorum quenching with a new application: Rotary microbial carrier frame. J. Membr. Sci..

[20] Nahm C.H., Choi D.C., Kwon H., Lee S., Lee S.H., Lee K., Choo K.-H., Lee J.-K., Lee C.-H., Park P.K. (2017). Application of quorum quenching bacteria entrapping sheets to enhance biofouling control in a membrane bioreactor with a hollow fiber module. J. Membr. Sci..

[21] Mideksa E., Teychene J., Soueid S., Laloo R., Fourquaux I., Castelain M., Sartor V., Tourrette A., Guigui C. (2025). Preparation and characterization of single-step sodium nitrate-calcium chloride crosslinked polyvinyl alcohol-alginate sheets for quorum quenching application in membrane bioreactors. J. Polym. Environ..

[22] Shah S.S.A., Lee K., Park H., Choo K.-H. (2022). Live membrane filters with immobilized quorum quenching bacterial strains for anti-biofouling. J. Membr. Sci..

[23] Pang H., Huang J., Li X., Yi K., Li S., Liu Z., Zhang W., Zhang C., Liu S., Gu Y. (2023). Enhancing quorum quenching media with 3D robust electrospinning coating: A novel biofouling control strategy for membrane bioreactors. Water Res..

[24] Kim S.-R., Oh H.-S., Jo S.-J., Yeon K.-M., Lee C.-H., Lim D.-J., Lee C.-H., Lee J.-K. (2013). Biofouling Control with Bead-Entrapped Quorum Quenching Bacteria in Membrane Bioreactors: Physical and Biological Effects. Environ. Sci. Technol..

[25] Kouassi G.K., Irudayaraj J., McCarty G. (2005). Examination of cholesterol oxidase attachment to magnetic nanoparticles. J. Nanobiotechnol..

[26] Ivanova V., Petrova P., Hristov J. (2011). Application in the ethanol fermentation of immobilized yeast cells in matrix of alginate/magnetic nanoparticles, on chitosan-magnetite microparticles and cellulose-coated magnetic nanoparticles. arXiv.

[27] Kim H., Noori A., Kim M.H., Lee C., Ko J.H., Hwang B.K., Lee K., Oh H.S. (2024). Biofouling mitigation of a membrane bioreactor for industrial wastewater treatment by quorum quenching. J. Membr. Sci..

[28] Hoekstra A.Y., Chapagain A., Martinez-Aldaya M., Mekonnen M. (2009). Water Footprint Manual: State of the Art 2009.

[29] Eisenträger A., Vella D., Griffiths I.M. (2014). Particle capture efficiency in a multi-wire model for high gradient magnetic separation. arXiv.

[30] Tesanovic M., de Souza J.P., Bazant M.Z., Berensmeier S. (2025). Magnetic particle capture in high-gradient magnetic separation: A theoretical and experimental study. AIChE J..

[31] Dursun D., Jimenez J., Briggs A. (2012). Comparison of process alternatives for enhanced nutrient removal: Perspectives on energy requirements and costs. Proc. Water Environ. Fed..

[32] Fan W., Zhang X. (2025). Magnetic coconut shell biochar/sodium alginate composite aerogel beads for efficient removal of methylene blue from wastewater: Synthesis, characterization, and mechanism. Int. J. Biol. Macromol..

[33] Wang R., Zhu J., Bao Y., Su X., Xiao X., Dong F., Chen C., Fu H., Song C., Sun F. (2025). Application of quorum quenching bacteria for biofouling control in membrane bioreactors treating landfill leachate. Sep. Purif. Technol..

[34] Kalya E., Alver A. (2023). Determining the contribution of the wastewater treatment plant to the sustainable environment with water footprint indicators. Environ. Dev. Sustain..

[35] Kim H., Lee C., Kim M.H., Choi G.E., Cayetano R.D.A., Hwang B.K., Oh H.S. (2025). Quorum quenching for biofouling control in a pilot-scale membrane bioreactor treating display manufacturing wastewater. Bioresour. Technol..

